# OPTICAL COHERENCE TOMOGRAPHY ANGIOGRAPHY FINDINGS OF MICROVASCULAR AND NEURAL CHANGES IN PRIMARY PULMONARY HYPERTENSION

**DOI:** 10.1097/IAE.0000000000002940

**Published:** 2020-08-05

**Authors:** Simin Gu, Zijing Li, Yichi Zhang, Yingmei Liu, Peng Zeng, Rui Zeng, Wenhui Wang, Jianhui Xiao

**Affiliations:** * Department of Ophthalmology, Sun Yat-Sen Memorial Hospital, Sun Yat-Sen University, Guangzhou, People's Republic of China; and; † Guangdong Provincial Key Laboratory of Malignant Tumor Epigenetics and Gene Regulation, Sun Yat-Sen Memorial Hospital, Sun Yat-Sen University, Guangzhou, People's Republic of China.

**Keywords:** primary pulmonary hypertension, optical coherence tomography angiography, ocular complications, retina, retinal nerve fiber layer, choroidal thickness

## Abstract

To investigate the microvascular and neural changes in primary pulmonary hypertension (PPH) patients compared with healthy controls.

**Methods:**

Forty-four eyes of 22 PPH patients were included in this observational clinical cohort study, and 44 eyes of 22 healthy participants were enrolled as controls. Optical coherence tomography angiography images were obtained from each participant using the RTVue XR Avanti device with AngioVue software 2.0.

**Results:**

Regarding the total macular-associated vessel density, including that of the superficial and deep retina, the optic disk–associated capillary density, including that of the whole image, capillary density inside the disk, and the peripapillary region, was significantly lower in the PPH group than in the control group. There was a similar trend in the retinal nerve fiber layer thickness and the ganglion cell complex thickness, whereas the focal loss volume and the global loss volume were greater in the PPH group than the control group.

**Conclusion:**

Changes in the capillary density and thickness of the retina and the optic nerve head in PPH patients can be detected by optical coherence tomography angiography. Parameters including the macular-associated vessel density, optic disk–associated capillary density, retinal nerve fiber layer, ganglion cell complex, focal loss volume, and global loss volume may provide useful evidence for the early detection of microvascular and neural impairments in patients with PPH.

## Abstract 2

In this study, we found that changes of the retina and optic nerve head in primary pulmonary hypertension can be detected by optical coherence tomography angiography. Main parameters may provide useful evidence for the early detection of ocular impairments in patients with primary pulmonary hypertension.

The sixth World Symposium on Pulmonary Hypertension Task Force proposed that precapillary pulmonary hypertension (precapillary PH) is best defined by the concomitant presence of mean pulmonary artery pressure of >20 mmHg, pulmonary artery wedge pressure of ≤15 mmHg, and pulmonary vascular resistance of ≥3 wood units. Pulmonary hypertension can be classified into different subtypes according to the clinical symptoms, pathological findings, hemodynamic characteristics, and treatment. It may be idiopathic or secondary to other clinical diseases.^1^

Ocular abnormalities are considered to be strongly associated primary PH (PPH), a subtype that accounts for approximately 10% of PH.^2^ The increase pressure in pulmonary artery leads to an increase in the superior vena cava venous pressure, which elevates the ocular venous pressure, resulting in ocular venous dilation and choroidal congestion; in turn, this leads to stasis of the ocular capillary network, ultimately resulting in serious ocular complications secondary to PPH, including ocular signs, such as proptosis,^3^ chemosis, dilated and tortuous episcleral vessels,^4^ and fundus abnormalities, such as hemorrhage, macular edema, central serous chorioretinopathy-like (CSC-like) changes,^5^ and secondary glaucoma.^6,7^ When PPH patients are referred to the clinic, most ocular abnormalities are asymptomatic or nonspecific.^8^ Traditional ophthalmic examinations, such as determination of the best-corrected visual acuity or intraocular pressure (IOP) and dilated fundus examination, do not have enough power to detect minor changes in the fundus. Fluorescein fundus angiography is one of the most useful examinations for diagnosing and assessing the severity of fundus diseases. However, it is an invasive technique and may not only cause severe anaphylaxis, even in some healthy individuals,^9^ but also does not allow the quantification of vessels. Optical coherence tomography angiography (OCTA), a noninvasive cross-sectional imaging technique, is frequently applied.^10^ High-resolution images can be captured and subtle capillary alterations can be distinguished on OCTA, making quantification feasible.^11,12^ In addition, some subtle changes in the preclinical stage can be detected, and the lesions can be located. However, there have been no reports of ocular abnormality analysis in PPH by OCTA.

The purpose of this hospital-based study was to report the signs of ocular abnormalities in PPH patients and changes in the capillary density (CD) of the retina and optic nerve head (ONH), the retinal nerve fiber layer (RNFL) thickness, and the ganglion cell complex (GCC) thickness between PPH patients and control subjects on OCTA.

## Methods

### Subjects

This observational clinical cohort study was approved by the research ethics committee of Sun Yat-Sen Memorial Hospital, Sun Yat-Sen University and adhered to the tenets of the Declaration of Helsinki. Forty-four eyes of 22 PPH subjects were included. After a dilated fundus examination by a very experienced ophthalmologist (the examiner and the subjects were double blinded), 4 PPH patients with fundus changes were included in Group 1 and 18 PPH patients without fundus changes were included in Group 2. Another 44 eyes of 22 healthy participants were included in Group 3 as controls. All subjects enrolled were patients at the Sun Yat-Sen Memorial Hospital from July 2017 to January 2019. The whole process was performed in the hospital, and informed consent was obtained from all study participants. The inclusion criteria included a diagnosis of PPH confirmed by a qualified cardiologist from the Sun Yat-Sen Memorial Hospital. All of the subjects enrolled had never used drugs that might induce PH or other known medicines that might influence ocular function. One subject with severe PPH had been treated with sildenafil to control the disease for more than 10 years.

Baseline ophthalmologic examinations could play a significant role in excluding primary ocular region illness. Ophthalmologic examinations, including determination of the best-corrected visual acuity or IOP and slit-lamp and dilated fundus examinations, were performed on the eyes. Color fundus photographs were obtained (7F-ETDRS) (Canon, Inc, Tokyo, Japan), the central corneal thickness, corneal curvature, anterior chamber depth, and axial length were measured using an IOL Master 700R system (Carl Zeiss Meditec AG, Jena, Germany); the visual field was evaluated with a Humphrey Field Analyzer (Central 30-2 Threshold Test), and OCTA was conducted (Optovue, Inc, Fremont, CA). Basic characteristics, including sex, age, medical history, PPH duration, mean pulmonary artery pressure, and treatment, were collected. The exclusion criteria were as follows: (1) primary ocular disease and (2) known systemic or other predictable factors that might result in ocular complications, such as diabetes, hypertension, and autoimmune disease. The healthy controls underwent the same ophthalmic examination process.

### Optical Coherence Tomography Angiography Examinations

Optical coherence tomography angiography images were obtained using the RTVue XR Avanti OCTA device (Optovue, Inc) with its prototype AngioVue software 2.0. The device operates at a speed of 70,000 A scans per second, a wavelength of 840 nm, and a frequency bandwidth of 45 nm. The split-spectrum amplitude-decorrelation angiography algorithm allows the OCTA device to detect erythrocyte movement noninvasively in real time. Centered on the fovea, both the 300-*µ*m-wide region and 600-*μ*m-wide region of the macula were scanned. The inner retinal and outer retinal vascular structures were observed in the screening; parameters in the same layer shared the same vascular plexus, and they had interactions with each other. HD Angio Disc 4.5-mm mode was used to capture a 4.5 mm × 4.5 mm area surrounding the optic disk. The area was divided into different sections, and the radial peripapillary capillary (RPC) density in these sections and the peripapillary thickness were automatically calculated by the software AngioVue 2.0. Furthermore, ONH mode was used to assess the images of the macula and the optic nerve fiber. Ganglion cell complex mode was used to measure the thickness of the RNFL, ganglion cell layer, and inner plexiform layer, and all OCTA examinations were performed by two very experienced ophthalmic examiners. The two examiners were doubled blinded to the subjects. Optical coherence tomography angiography images with a scan quality of <6 were excluded.

## Results

### Patient Characteristics

Forty-four eyes of 22 subjects and 44 eyes of 22 healthy controls were enrolled in this study. There was no statistical significance in age or sex between the PPH and control subjects. Regarding the baseline ophthalmologic examination, there were statistically significant differences in the best-corrected visual acuity and IOP between Group 1 and Groups 2 and 3 (numerical variables were compared among the three groups [Group 1: PPH with fundus changes, Group 2: PPH without fundus changes, and Group 3: healthy controls] by one-way analysis of variance). The characteristics of the participants, including the mean age, sex, corneal thickness, anterior chamber depth, and axial length, were not significantly different among the three groups. There was a significant increase in the mean pulmonary artery pressure in Group 1 compared with Group 2, but there was no significant difference in the PPH duration between the two cohorts. The characteristics of the participants are presented in Table 1.

**Table 1.** Patient Characteristics

Items	Group 1	Group 2	Group 3	Group 1 Versus Group 2, *P*	Group 2 Versus Group 3, *P*	Group 1 Versus Group 3, *P*
Eyes (n)	8	36	44	NA	NA	NA
Mean age, range (years)	42.38 ± 16.58	44.13 ± 16.949	41.75 ± 17.302	0.838	0.782	0.942
Male: female	2:2	7:11	11:11	NA	NA	NA
PPH duration (years)	5.125 ± 4.324	3.125 ± 2.167	NA	0.066	—	—
mPAP (mmHg)	79.125 ± 21.304	53.500 ± 15.464	NA	0.016	NA	NA
BCVA (logMAR [Snellen])	0.363 ± 0.151 (20/33)	0.089 ± 0.135 (20/20)	−0.043 ± 0.695 (20/15)	0.000	0.000	0.000
Corneal thickness (*μ*m)	555.13 ± 31.037	573.25 ± 32.849	543.88 ± 20.794	0.221	0.441	0.054
Anterior chamber depth (mm)	3.4038 ± 0.305	3.0313 ± 0.500	3.480 ± 0.605	0.140	0.079	0.757
Axial length (mm)	23.089 ± 0.781	24.266 ± 0.564	23.534 ± 1.728	0.052	0.216	0.445
IOP (mmHg)	28.50 ± 14.162	16.25 ± 2.121	15.00 ± 1.309	0.008	0.776	0.004

BCVA, best-corrected visual acuity; Group 1, PPH with fundus changes; Group 2, PPH without fundus changes; Group 3, control; mPAP, mean pulmonary arterial pressure; logMAR, logarithm of the minimum angle of resolution; NA, not available.

*P* < 0.05.

The ocular symptoms reported by the PPH patients with fundus changes were nonspecific and included blurred vision, metamorphopsia as a result of macular involvement, severe eye pain resulting from high IOP, and red eyes because of vascular changes.^7,13^ Anterior segment manifestations, such as chemosis and dilated and tortuous conjunctival and episcleral veins, are common, and they can be observed not only in PPH patients with fundus changes but also occasionally in PPH patients without fundus changes. Other signs, such as corneal edema, could be assessed in Group 1.^14^ Moreover, fundus abnormalities, including tortuous retinal vessels, retinal hemorrhage, macular edema, and CSC-like changes, were observed during the dilated fundus examination in Group 1 subjects.^15^ In our cases, the main ocular manifestations are shown in Table 2.

**Table 2.** Major Ocular Signs

Ocular Signs	N (%)
Intraocular hypertension	11.36% (5/44)
Chemosis	6.82% (3/44)
Dilated and tortuous episcleral vessel	36.36% (16/44)
Fundus abnormality	
Dilated and tortuous retinal vessels	18.18% (8/44)
Retinal hemorrhage	18.18% (8/44)
macular edema	2.27% (1/44)
Macular neuroepithelial detachment	2.27% (1/44)

### Optical Coherence Tomography Angiography Findings

The parameters compared among Groups 1, 2, and 3 included the foveal avascular zone, macular-associated vessel density (VD), optic disk–associated CD, GCC thickness, RNFL thickness, focal loss volume (FLV), global loss volume (GLV), subfoveal choroidal thickness, and choriocapillary flow area. Statistical analysis of the OCTA data was performed using SPSS 25.0 (SPSS, Inc, Chicago, IL). The numerical variables were compared among the three groups by one-way analysis of variance, and Least Significant Difference post hoc analysis was performed to evaluate the significant differences.

**Fig. 1.**
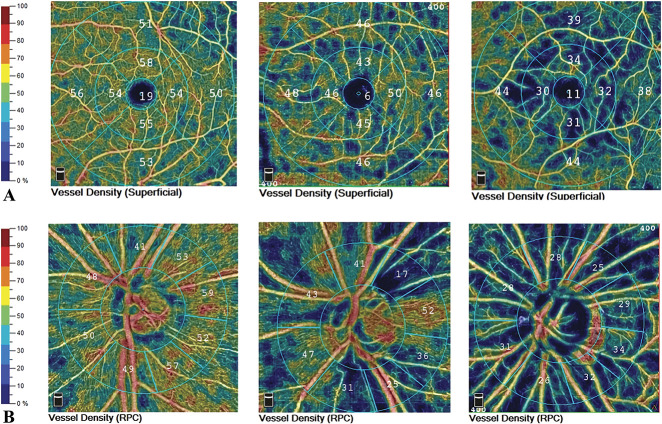
Ganglion cell complex and ONH reports. The GCC consists of three layers: the RNFL, the ganglion cell layer, and the inner plexiform layer. **A.** (left: GCC report; right: RNFL report): the right eye of a 25-year-old female control subject. **B.** (left: GCC report; right: RNFL report): the right eye of a 28-year-old female PPH patient without fundus changes. **C.** (left: GCC report; right: RNFL report): the right eye of a 28-year-old female PPH patient with fundus changes.

Regarding the total macular-associated VD, including the superficial and deep retina, the optic disk–associated CD, including that of the whole image, inside the disk, and in the peripapillary region, was significantly reduced in Group 1 compared with Group 3 (superficial total VD: 38.373 ± 6.926% vs. 44.975 ± 4.984%; *P* = 0.003; deep total VD: 45.1400 ± 3.638% vs. 50.184 ± 3.980%; *P* = 0.003; CD inside the disk (ring diameter = 4.5 mm): 54.170 ± 11.736 vs. 61.984 ± 3.546%; *P* = 0.000; peripapillary CD (ring diameter = 4.5 mm) 47.055 ± 6.087 vs. 58.813 ± 2.338%; *P* = 0.000; whole RPC density: 45.788 ± 5.451 vs. 55.952 ± 2.656; *P* = 0.000). However, there was no significant difference in the foveal avascular zone or foveal VD (including the superficial and the deep retinal) between the two cohorts (foveal avascular zone: 2.327 ± 0.526 vs. 2.365 ± 0.289; *P* = 0.880; superficial foveal VD: 12.116 ± 8.409 vs. 14.825 ± 5.738; *P* = 0.156; deep foveal VD: 23.746 ± 11.246 vs. 29.780 ± 10.104; *P* = 0.114). The perifoveal VD in the superficial retina and the parafoveal and perifoveal VD in the deep retina were lower in Group 2 than Group 3 (superficial perifoveal VD: 46.548 ± 5.781 vs. 50.242 ± 3.901; *P* = 0.001; deep parafoveal VD: 52.550 ± 4.332 vs. 53.000 ± 4.102; *P* = 0.017; deep perifoveal VD: 46.652 ± 6.162 vs. 50.033 ± 7.707; *P* = 0.035). There was a similar in the RPC and optic disk–associated CD when comparing Groups 2 and 3 (Figure 1).

The RNFL thickness was significantly reduced in Group 1 compared with Groups 2 and 3 (Group 1 vs. Group 2: 79.42 ± 22.56 vs. 98.491 ± 11.100; *P* = 0.000; Group 1 vs. Group 3: 79.42 ± 22.56 vs. 101.338 ± 11.249; *P* = 0.000). Additionally, the GCC thickness was reduced in Group 1 (Group 1 vs. Group 2: 79.349 ± 18.589 vs. 96.609 ± 14.658; *P* = 0.001; Group 1 vs. Group 3: 79.349 ± 18.589 vs. 97.450 ± 9.370, *P* = 0.000). Furthermore, the FLV and GLV were increased in Group 1 compared to the other two groups (FLV: Group 1 vs. Group 2: 7.072 ± 5.955 vs. 1.544 ± 2.703, *P* = 0.000, Group 1 vs. control: 7.072 ± 5.955 vs. 0.972 ± 0.766, *P* = 0.000; GLV: Group 1 vs. Group 2: 19.632 ± 15.174 vs. 4.590 ± 6.785, *P* = 0.001, Group 1 vs. control: 19.632 ± 15.174 vs. 3.019 ± 3.934; *P* = 0.001). Moreover, the differences in the macular-associated VD, optic nerve–associated CD, RNFL thickness, GCC thickness, FLV, and GLV between Groups 1 and 2 were statistically significant. Details are shown in Tables 3 and 4.

**Table 3.** Major VD OCTA Measurements in the PPH Patients and Control Subjects

Items	Group 1	Group 2	Group 3	Group 1 Versus Group 2, *P*	Group 1 Versus Group 3, *P*	Group 2 Versus Group 3, *P*
Total VD (%)						
Superficial total VD (ILM-IPL)	38.373 ± 6.926	43.733 ± 6.168	44.975 ± 4.984	0.018	0.003	0.333
Foveal VD	12.116 ± 8.409	16.291 ± 8.958	14.825 ± 5.738	0.156	0.350	0.385
Parafoveal VD	41.848 ± 6.691	46.731 ± 6.247	47.950 ± 5.559	0.038	0.009	0.364
Temporal	34.707 ± 11.050	28.760 ± 15.502	46.766 ± 5.238	0.177	0.006	0.000
Superior	43.461 ± 6.666	47.279 ± 7.075	49.746 ± 5.713	0.130	0.013	0.091
Nasal	43.563 ± 7.184	46.182 ± 6.033	46.715 ± 5.830	0.091	0.027	0.179
Inferior	39.613 ± 8.104	48.083 ± 7.133	49.060 ± 5.729	0.001	0.000	0.509
Perifoveal VD	39.823 ± 4.995	46.548 ± 5.781	50.242 ± 3.901	0.001	0.000	0.001
Temporal	39.410 ± 5.483	46.210 ± 6.523	45.917 ± 4.494	0.002	0.003	0.817
Superior	40.886 ± 6.321	46.510 ± 5.750	50.591 ± 4.175	0.009	0.000	0.001
Nasal	45.130 ± 5.819	50.394 ± 6.188	54.240 ± 4.112	0.012	0.000	0.002
Inferior	40.378 ± 6.573	46.912 ± 6.686	50.229 ± 4.072	0.003	0.003	0.010
Deep total VD (IPL-OPL)	45.1400 ± 3.638	49.510 ± 4.694	50.184 ± 3.980	0.010	0.003	0.010
Foveal VD	23.746 ± 11.246	29.780 ± 10.104	29.056 ± 6.480	0.104	0.114	0.908
Parafoveal VD	48.504 ± 4.881	52.550 ± 4.332	53.000 ± 4.102	0.017	0.008	0.017
Temporal	48.149 ± 7.682	48.148 ± 7.682	53.250 ± 5.625	0.010	0.006	0.799
Superior	47.328 ± 4.979	47.328 ± 4.980	52.529 ± 4.800	0.015	0.009	0.778
Nasal	51.185 ± 5.814	51.185 ± 5.814	53.361 ± 4.911	0.264	0.276	0.938
Inferior	47.436 ± 6.382	51.465 ± 6.160	52.563 ± 4.488	0.060	0.016	0.369
Perifoveal VD	44.174 ± 5.170	46.652 ± 6.162	50.033 ± 7.707	0.363	0.031	0.035
Temporal	44.190 ± 7.100	48.546 ± 6.879	52.900 ± 6.200	0.097	0.001	0.005
Superior	43.868 ± 5.440	46.439 ± 7.083	50.181 ± 8.333	0.392	0.035	0.035
Nasal	44.966 ± 4.457	45.803 ± 6.897	48.444 ± 8.927	0.786	0.253	0.144
Inferior	43.945 ± 6.163	45.844 ± 9.193	48.610 ± 8.380	0.533	0.123	0.124
RPC density (%): (ILM-RNFL)						
Whole density (%)	45.788 ± 5.451	54.217 ± 3.606	55.952 ± 2.656	0.000	0.000	0.027
Whole capillary (%)	39.541 ± 6.725	47.910 ± 3.322	49.544 ± 2.447	0.000	0.000	0.035
CD inside disk (ring diameter = 4.5 mm) (%)	54.170 ± 11.736	58.926 ± 5.436	61.984 ± 3.546	0.030	0.000	0.017
Peripapillary CD (ring diameter = 4.5 mm) (%)	47.055 ± 6.087	56.492 ± 4.064	58.286 ± 3.149	0.000	0.000	0.045
CCF (mm^2^)	2.040 ± 0.394	2.002 ± 0.199	2.102 ± 0.100	0.772	0.452	0.641

CD, optic disk–associated capillary density; CCF, choriocapillary flow area; Group 1, PPH with fundus changes; Group 2, PPH without fundus changes; Group 3, control; ILM, internal limiting membrane; IPL, inner plexiform layer; OPL, outer plexiform layer; RPC, radial peripapillary capillaries; VD, macular-associated vessel density.

*P* < 0.05.

**Table 4.** Retinal nerve fiber layer, GCC, FLV, GLV, and SFCT Measurements in the PPH Patients and Control Subjects

	Group 1	Group 2	Group 3	Group 1 Versus Group 2, *P*	Group 1 Versus Group 3, *P*	Group 2 Versus Group 3, *P*
RNFL	79.42 ± 22.559	98.491 ± 11.100	101.338 ± 11.249	0.000	0.000	0.324
GCC	79.349 ± 18.589	96.609 ± 14.658	97.450 ± 9.370	0.001	0.000	0.770
FLV	7.072 ± 5.955	1.544 ± 2.703	0.972 ± 0.766	0.000	0.000	0.314
GLV	19.632 ± 15.174	4.590 ± 6.785	3.019 ± 3.934	0.001	0.001	0.296
SFCT	411.625 ± 91.206	389.130 ± 49.007	289.380 ± 55.666	0.311	0.000	0.000

Group 1, PPH with fundus changes; Group 2, PPH without fundus changes; Group 3, control; SFCT, subfoveal choroidal thickness.

*P* < 0.05.

## Discussion

Pulmonary hypertension ultimately remains a fatal disease if left untreated and could result in progressive right ventricular failure and even death because of the increase in venous pressure. Additionally, the increasing superior vena cava pressure elevates the ocular venous pressure, resulting in ocular venous dilation and choroidal congestion; in turn, this leads to stasis of the ocular capillary network and ultimately results in serious ocular abnormalities secondary to PPH, including ocular signs, such as proptosis, chemosis, dilated and tortuous episcleral vessels, corneal edema, and fundus abnormalities, such as hemorrhage, macular edema, CSC-like changes, and secondary glaucoma. Moreover, ciliary detachment, uveal effusion, exudative retinal detachment, central retinal vein occlusion,^7^ and branch retinal vein occlusion^16^ associated with PH have been reported.

Optical coherence tomography angiography, which allows the noninvasive assessment and measurement of vascular structures in the retina and ONH, is now a widely accepted ophthalmic imaging technique for examining the ocular vasculature.^11^ This tool, with good repeatability and reproducibility, provides useful information in terms of the VD, ocular blood flow, and thickness of the ONH and retina.^10,17^ Optical coherence tomography angiography enables visualization of the blood flow in the retina and ONH without the intravenous injection of dye, and it may thus be safer and more efficient than other methods.^18^ Optical coherence tomography angiography can feasibly be used for frequent assessments and screening in the initial stages of PPH in daily clinical practice and to detect changes in the ONH and retina. In our study, significant reductions in the VD of the retina and ONH and the thickness of the GCC and RNFL were observed in PPH, which have not been reported in previous research. Additionally, there have been no OCTA studies quantifying changes in the ONH and retina in PPH.

In our present study, PPH subjects and healthy control subjects were included, and a reduced total VD (including the superficial total VD and the deep total VD) was observed in the PPH patients compared with the control subjects. The parafoveal VD and the perifoveal VD (including superficial and deep) were obviously decreased in Groups 1 and 2 compared with Group 3. The macular-associated VD seemed to be influenced in PPH secondary to the increasing PH. Furthermore, there was a significant difference between Groups 1 and 2. It needs to be emphasized that changes in the macular-associated VD occurred in the preclinical stage before ocular symptoms and signs occur in PPH. Group 1 showed a decrease in the foveal VD in both the superficial and deep layers; unexpectedly, there was a mildly increasing trend in the foveal VD of Group 2 (including superficial and deep) compared with Group 3. In the early stage of the disease, PPH involves peripheral vascular resistance, and the resistance and vascular remodeling in the periphery may cause compensatory changes in other vascular beds, including the vessels in macula,^19^ to maintain its visual function. However, occlusive disease or possibly pathological changes may signal a future occlusive event and ultimately result in reduced ocular perfusion in the fundus in PPH.^20^

**Fig. 2.**
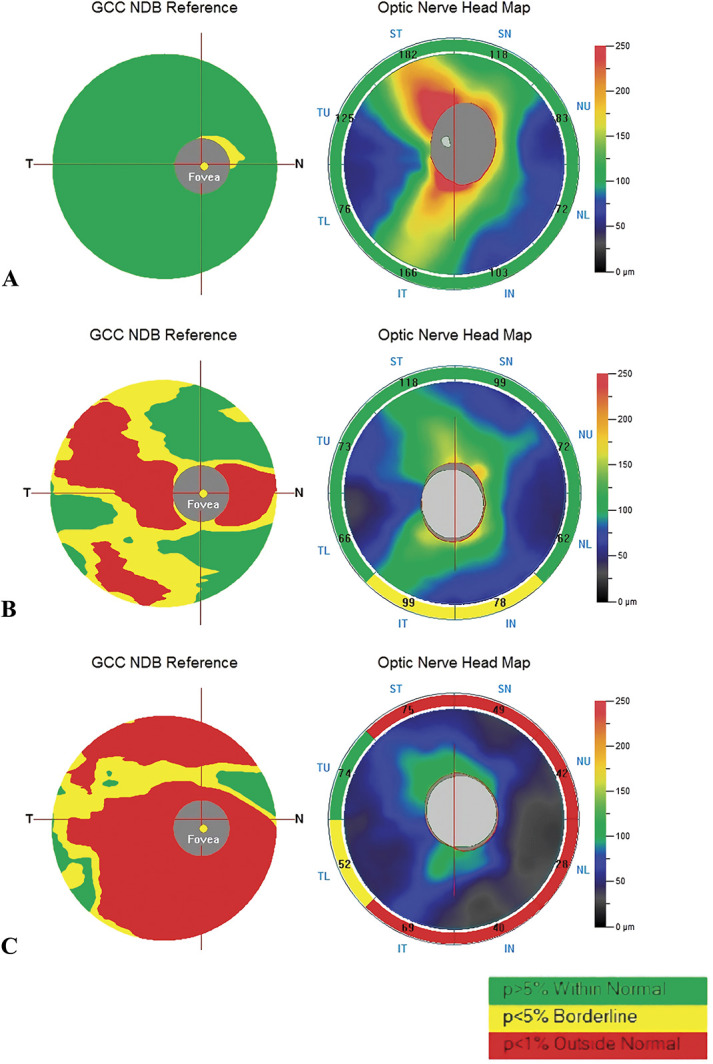
Superficial VD (macular-associated VD) and deep VD (optic disk–associated CD) report: (**A**): left: the left eye of a 25-year-old female control subject; middle: the left eye of a 28-year-old female PPH patient without fundus changes, right: the left eye of a 28-year-old female PPH patient with fundus changes. **B.** left: the left eye of a 25-year-old female control subject, middle: the left eye of a 28-year-old female PPH patient without fundus changes, right: the left eye of a 28-year-old female PPH patient with fundus changes.

In our study, the optic disk–associated CD, including the whole RPC density, the CD inside the disk, and the peripapillary CD, was significantly reduced in the PPH group compared with the control group. Furthermore, a significantly decreasing trend in the optic disk–associated CD was also observed in Groups 1 and 2. Therefore, the chronic retinal hypoxia caused by the stasis resulting from the increased pressure in the choroidal circulation that is induced by increasing pulmonary arterial hypertension may not only contribute to vascular dropout and remodeling in the macular area but also affect the capillaries in the disk. Similarly, neural impairment was noted (Figure 2). A significant decrease in the RNFL thickness and the GCC thickness, and an increase in the FLV and GLV were found in the PPH patients (including those in Groups 1 and 2). In our study, five eyes showed intraocular hypertension in Group 1 and were diagnosed with open-angle glaucoma secondary to pulmonary arterial hypertension after inspection (including gonioscopy). It must be stressed that elevations in IOP are mainly caused by blocking of the aqueous humor outflow pathway, and the difference between the IOP and the episcleral venous pressure provides the force to allow the aqueous humor to flow out of the trabecular meshwork.^21^ In PPH, increased ocular venous pressure secondary to increased pulmonary arterial pressure results in elevated episcleral venous pressure, which blocks the aqueous humor outflow pathway and eventually induces secondary glaucoma.

Many published studies have indicated that the CD inside the disk and the peripapillary region are significantly reduced in open-angle glaucoma and that the RNFL thickness and the GCC thickness are decreased.^22,23^ Studies have shown that pathological IOP is a major cause of retinal ganglion cell and optic nerve impairment^28^; increased IOP can not only directly cause retinal ganglion cell death but also lead to optic neuropathy resulting from ischemia.^24^ However, a trend toward a reduced RNFL and GCC thickness was also observed in Group 2 despite the absence of increased IOP. This suggests that increased IOP may not be a major risk factor for structural changes in the optic nerve in PPH; however, poor circulation in the retina and optic nerve is a main risk factor for structural injury progression. The blood supply of the optic nerve was decreased in the PPH patients, ultimately leading to damage. Therefore, optic nerve impairment started early in PPH even without clinical symptoms, and the ultimate increase in IOP could worsen the optic nerve damage.

In our study, a significantly greater subfoveal choroidal thickness was observed in the PPH subjects. Increased ocular venous pressure is associated with elevated systemic venous pressure, which affects ocular hemodynamics and causes blood stasis, resulting in thickening of the choroid, and an increased choroidal thickness is a risk factor for CSC. Primary pulmonary hypertension patients have an increased choroidal thickness, and the incidence of CSC among these patients is not unusual.^25^ In addition, sildenafil, a frequently used drug to control PPH, was found to increase the choroidal blood flow and choroidal thickness.^26^ Central serous chorioretinopathy occurring after receiving treatment with sildenafil has been previously reported.^27,28^ Thus, PPH patients treated with sildenafil are more likely to develop CSC. In our patients (Group 1: one patient had been treated with sildenafil for more than 10 years), CSC associated with sildenafil was observed. However, some results in our study are somewhat unexpected considering such preconceptions because the choriocapillary flow was not significantly different between the groups. The higher content of melanocyte pigment in the choroid and its location below the retinal pigment epithelium may influence the measurement outcomes of OCTA. Therefore, future studies are needed to clarify the possible mechanism underlying this finding.

In conclusion, preclinical ocular changes in PPH can be detected by OCTA. Parameters such as the macular-associated VD, optic disk–associated CD, RNFL thickness, and GCC thickness may be useful for the early detection of microvascular and neural impairments in PPH patients with or without fundus changes. Moreover, the deep VD, including the parafoveal and perifoveal VD, and the RNFL and GCC thickness, may be sensitive predictors of ocular impairment in PPH patients without fundus abnormities. This is also the first report to quantify and evaluate vascular dropout and neural impairment in PPH patients.
